# Sensitization of dural afferents underlies migraine-related behavior following meningeal application of interleukin-6 (IL-6)

**DOI:** 10.1186/1744-8069-8-6

**Published:** 2012-01-24

**Authors:** Jin Yan, Ohannes K Melemedjian, Theodore J Price, Gregory Dussor

**Affiliations:** 1 Department of Pharmacology, University of Arizona College of Medicine, 1501 N Campbell Ave, PO Box 245050, Tucson, AZ 85724, USA; 2 Graduate Interdisciplinary Program in Neuroscience, University of Arizona, 1548 E. Drachman St., PO Box 210476, Tucson, AZ 85719, USA; 3 Bio5 Institute, University of Arizona, 1657 E Helen St., PO Box 210240, Tucson, AZ 85721, USA

**Keywords:** Migraine, Nav1.7, Interleukin-6, Dural afferents, Meninges, Pain, Headache

## Abstract

**Background:**

Migraine headache is one of the most common neurological disorders, but the pathophysiology contributing to migraine is poorly understood. Intracranial interleukin-6 (IL-6) levels have been shown to be elevated during migraine attacks, suggesting that this cytokine may facilitate pain signaling from the meninges and contribute to the development of headache.

**Methods:**

Cutaneous allodynia was measured in rats following stimulation of the dura with IL-6 alone or in combination with the MEK inhibitor, U0126. The number of action potentials and latency to the first action potential peak in response to a ramp current stimulus as well as current threshold were measured in retrogradely-labeled dural afferents using patch-clamp electrophysiology. These recordings were performed in the presence of IL-6 alone or in combination with U0126. Association between ERK1 and Nav1.7 following IL-6 treatment was also measured by co-immunoprecipitation.

**Results:**

Here we report that in awake animals, direct application of IL-6 to the dura produced dose-dependent facial and hindpaw allodynia. The MEK inhibitor U0126 blocked IL-6-induced allodynia indicating that IL-6 produced this behavioral effect through the MAP kinase pathway. In trigeminal neurons retrogradely labeled from the dura, IL-6 application decreased the current threshold for action potential firing. In response to a ramp current stimulus, cells treated with IL-6 showed an increase in the numbers of action potentials and a decrease in latency to the first spike, an effect consistent with phosphorylation of the sodium channel Nav1.7. Pretreatment with U0126 reversed hyperexcitability following IL-6 treatment. Moreover, co-immunoprecipitation experiments demonstrated an increased association between ERK1 and Nav1.7 following IL-6 treatment.

**Conclusions:**

Our results indicate that IL-6 enhances the excitability of dural afferents likely via ERK-mediated modulation of Nav1.7 and these responses contribute to migraine-related pain behavior *in vivo*. These data provide a cellular mechanism by which IL-6 in the meninges causes sensitization of dural afferents therefore contributing to the pathogenesis of migraine headache.

## Background

Migraine is characterized as episodes of unilateral throbbing headache accompanied by a variety of symptoms, including aura, nausea, vomiting, photophobia and phonophobia [1]. Although the mechanisms contributing to migraine pathophysiology are not fully known, one hypothesis proposes that migraine is an inflammatory disease [2]. This idea is supported by the efficacy of non-steroidal anti-inflammatory drugs (NSAIDs) in migraine therapy as well as increased intracranial levels of inflammatory mediators during migraine attacks [2,3]. Interleukin-6 (IL-6), which is one such mediator found to be elevated during migraine attacks [3,4], is a cytokine with an established role in modulating various inflammatory pain conditions, including skin incision, carrageenan injection, burn-injury pain and pancreatitis-induced pain [5-9]. IL-6 levels are increased under inflammatory conditions and increases in IL-6 parallel pain intensity over time [10-12]. IL-6's pain promoting actions are thought to be mediated by a direct action on nociceptors because sensory neuron specific knockout of the IL-6 co-receptor reduces nociceptive sensitization [13]. Moreover, neutralizing IL-6 using a monoclonal antibody is effective in treating human rheumatoid arthritis [14]. Thus, accumulating evidence points to IL-6 as a contributing factor to many pain conditions possibly including migraine.

Activation and sensitization of meningeal nociceptors leads to afferent signaling that is thought to contribute to the headache that occurs during migraine. However, the contribution of IL-6 to this process and the mechanisms by which this may occur have not yet been explored. Following acute IL-6 application, trigeminal ganglion neurons display phosphorylation of ERK [15] indicating that these neurons respond to IL-6 through activation of the Mitogen-Activated Protein Kinase (MAPK) signaling pathway. Activation of the ERK1/2 MAPK pathway has been implicated in induction and maintenance of various pain conditions via transcriptional, translational or post-translational regulation [15-17]. Recent work has identified the voltage-gated sodium channel Nav1.7 as a novel downstream post-translational target for MAPK. Nav1.7 is a threshold sodium channel expressed on small and medium DRG neurons [18] and inhibition of ERK1/2 decreased neuronal excitability by inhibiting Nav1.7 phosphorylation and altering its gating properties [19].

Taken together, these studies led us to propose that increased levels of IL-6 in the meninges produces migraine-related pain behavior and this hypothesis was addressed using a preclinical model of headache. Further, we examined whether dural afferent excitability was increased following IL-6 exposure and whether this increased excitability is consistent with sodium channel phosphorylation.

## Methods

### Animals

Adult male Sprague Dawley rats (175-200 g) were maintained in a climate-controlled room on a 12 h light/dark cycle with food and water ad libitum. All procedures were performed in accordance with the policies and recommendations of the International Association for the Study of Pain, the National Institutes of Health guidelines for the handling and use of laboratory animals, and were approved by the Institutional Animal Care and Use Committee of the University of Arizona.

### Surgical preparation

1. Tracer injection

Dural afferents were identified as previously described [20,21]. Seven days prior to sacrifice, animals were anesthetized with a combination of ketamine and xylazine (80 mg/kg and 12 mg/kg; Sigma-Aldrich). Two holes were made in the skull under a dissecting microscope to carefully expose but not damage the dura and fluorogold (5 μl/hole; 4% in SIF, synthetic interstitial fluid, pH 7.4, 320 mOsm) was applied onto the dura. A small piece of gelfoam was retained in the hole to increase the absorption of the dye and prevent spread of the tracer outside of the hole. Holes were covered with bone wax to prevent tracer spread. The incision was closed with sutures. Immediately postoperatively, animals received a single subcutaneous injection of gentamicin (8 mg/kg) to minimize infection. Undamaged dura at the injection sites was evaluated at the time the animals were sacrificed and only animals with intact dura and no signs of damage were used for further experiments.

2. Dural cannulation

Dura cannulae were implanted as previously described [20,21]. Animals were anesthetized with a combination of ketamine and xylazine (80 mg/kg and 12 mg/kg; Sigma-Aldrich). A 2 cm incision was made to expose the skull. A 1 mm hole was made in the skull (above the transverse sinus; 2 mm left of the sagittal suture and 2 mm anterior to the lambdoid suture) with a hand drill (DH-0 Pin Vise; Plastics One, Roanoke, VA) to carefully expose the dura. A guide cannula (22 GA, #C313G; Plastics One), designed to extend 0.5 mm from the pedestal to avoid irritation of the dural tissue, was inserted into the hole and sealed into place with glue. Two additional 1 mm holes were made in the parietal bones to receive stainless-steel screws (Small Parts), and dental acrylic was used to fix the cannula to the screws. A dummy cannula (#C313DC; Plastics One) was inserted to ensure patency of the guide cannula. Immediately postoperatively, animals received a single subcutaneous injection of gentamicin (8 mg/kg) to minimize infection. Rats were housed separately and allowed 6 to 8 days of recovery.

### Cell culture

1. Whole-cell patch clamp

Seven days following fluorogold application, trigeminal ganglia were removed, enzymatically treated, and mechanically dissociated as previously described [21]. Rats were anesthetized with isoflurane (Phoenix Pharmaceuticals) and sacrificed by decapitation. The trigeminal ganglion (TG) were removed and placed in ice-cold Hanks balanced-salt solution (divalent free). Ganglia were cut into small pieces and incubated for 25 mins in 20 U/ml Papain (Worthington) followed by 25 mins in 3 mg/ml Collagenase TypeII (Worthington). Ganglia were then triturated through fire-polished pasteur pipettes and plated on poly-D-lysine (Becton Dickinson) and laminin (Sigma)-coated plates. After several hours at room temperature to allow adhesion, cells were cultured in a room-temperature, humidified chamber in Liebovitz L-15 medium supplemented with 10% FBS, 10 mM glucose, 10 mM HEPES and 50 U/ml penicillin/streptomycin. Cells were used within 24 h post plating.

2. Western Blotting

Rat trigeminal ganglia (TG) were excised aseptically and placed in Hank's Buffered Salt Solution (HBSS, Invitrogen) on ice. The ganglia were dissociated enzymatically with collagenase A (1 mg/ml, 25 min, Roche) and collagenase D (1 mg/ml, Roche) with papain (30 U/ml, Roche) for 20 min at 37°C. To eliminate debris, 70 μm (BD) cell strainers were used. The dissociated cells were resuspended in DMEM/F12 (Invitrogen) containing 1X pen-strep (Invitrogen), 1X GlutaMax, 3 μg/ml 5-FDU (5-Fluoro-2'-deoxyuridine) (Sigma), 7 μg/ml uridine (Sigma) and 10% fetal bovine serum (Hyclone). The cells were plated in 6-well plates (BD Falcon) and incubated at 37°C in a humidified 95% air/5%CO2 incubator. On day 5 the cells were washed in DMEM/F12 media for 15 mins followed by treatment.

### Electrophysiology

Whole cell patch-clamp experiments were performed on isolated rat TG using a MultiClamp 700B (Axon Instruments) patch-clamp amplifier and pClamp 10 acquisition software (Axon Instruments). Recordings were sampled at 2 kHz and filtered at 1 kHz (Digidata 1322A, Axon Instruments). Pipettes (OD: 1.5 mm, ID: 0.86 mm, Sutter Instrument) were pulled using a P-97 puller (Sutter Instrument) and heat polished to 2.5-4 MΩ resistance using a microforge (MF-83, Narishige). Series resistance was typically < 7 MΩ and was compensated 60-80%. All recordings were performed at room temperature. A Nikon TE2000-S Microscope equipped with a mercury arc lamp (X-Cite^® ^120) was used to identify FG-labeled dural afferents. Data were analyzed using Clampfit 10 (Molecular Devices) and Origin 8 (OriginLab). Cell sizes were not significantly different among groups (Vehicle: 38.91 ± 2.214 pF vs IL-6: 39.62 ± 1.712 pF vs IL-6 + U0126: 41.98 ± 2.526 pF, *p *> 0.05). Pipette solution contained (in mM) 140 KCl, 11 EGTA, 2 MgCl_2_, 10 NaCl, 10 HEPES, 1 CaCl_2 _pH 7.3 (adjusted with N-methyl glucamine), and was ~ 320 mosM. External solution contained (in mM) 135 NaCl, 2 CaCl_2_, 1 MgCl_2_, 5 KCl, 10 Glucose, 10 HEPES, pH 7.4 (adjusted with N-methyl glucamine), and was ~ 320 mosM.

### Behavioral testing

Rats were acclimated to suspended Plexiglas chambers (30 cm long × 15 cm wide × 20 cm high) with a wire mesh bottom (1 cm2). Ten μl of vehicle or testing solution was injected through an injection cannula (28GA, #C313I; Plastics One) cut to fit the guide cannula. Withdrawal thresholds to probing the face and hind-paws were determined at 1-h intervals after administration. A behavioral response to calibrated von Frey filaments applied to the midline of the forehead, at the level of the eyes, was indicated by a sharp withdrawal of the head. Paw withdrawal (PW) thresholds were determined by applying von Frey filaments to the plantar aspect of the hind-paws, and a response was indicated by a withdrawal of the paw. The withdrawal thresholds were determined by the Dixon up-down method [22]. Maximum filament strengths were 8 and 15 g for the face and hind-paws, respectively.

### Western blotting

Protein was extracted from cells in lysis buffer (50 mM Tris HCl, 1% Triton X-100, 150 mM NaCl, and 1 mM EDTA at pH 7.4) containing protease and phosphatase inhibitor mixtures (Sigma) with an ultrasonicator on ice, and cleared of cellular debris and nuclei by centrifugation at 14,000 RCF for 15 min at 4°C. Fifteen micrograms of protein per well were loaded and separated by standard 7.5% or 10% SDS-PAGE. Proteins were transferred to Immobilon-P membranes. Blots were incubated with primary antibody overnight at 4°C and detected the following day with appropriate secondary antibodies. Signal was detected by ECL on chemiluminescent films. Densitometric analyses were performed with Image J software (NIH).

### Co-immunoprecipitation

After protein extraction, 70 μg protein was incubated with Nav1.7 antibody (NeuroMab) overnight at 4°C followed by 3 h incubation with 20 μl protein G-Sepharose beads. After washing 3 times with lysis buffer, the pelleted beads were resuspended in 1X Laemmli Sample Buffer containing 5% v/v β-mercaptoethanol and total ERK (tERK) bound to the precipitated beads was analyzed by western blotting.

### Data analysis

All data are presented as means ± SEM unless otherwise noted. Statistical evaluation was performed by linear regression analysis, unpaired t-test, one-way analysis of variance (ANOVA) followed by post hoc Newman-Keuls test, or two-way ANOVA. For behavioral experiments, data were converted to area over the time-effect curve to allow for multiple comparisons.

### Chemicals

Fluorogold was purchased from Fluorochrome, LLC. and dissolved in synthetic interstitial fluid (SIF) (pH 7.4, 310 mOsm) to 4%. U0126 was from Tocris Biosciences. Rat recombinant IL-6 was from R&D Systems. Stock U0126 (10 mM) was prepared in Dimethyl sulfoxide (DMSO) and added to the culture media and the recording chamber to produce a final concentration of 10 μM in 0.1% DMSO for patch experiment. For the behavioral experiments, stock U0126 solutions (100 mM in DMSO) were prepared and diluted in SIF to the final concentration of 1 mM. Vehicle control was SIF with 1% DMSO. Stock rIL-6 (10 μg/ml) was prepared in sterile 0.1% BSA in PBS and diluted to final concentrations of 50 ng/ml (patch) and 100 ng/ml (behavior), respectively.

## Results

### Cutaneous allodynia following IL-6 administration to the dura

**Figure 1:**
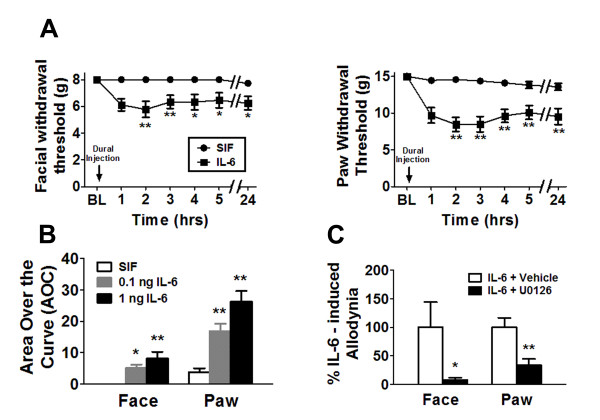
**Application of IL-6 to the dura elicited cutaneous allodynia via activation of the MEK/ERK pathway**. (**A**) Withdrawal thresholds to tactile stimuli applied to the face and the hind-paws were measured in rats before and immediately after dural application of 1 ng IL-6 (n = 16) or SIF (n = 17). For both facial and hind-paw responses, two-factor analysis of variance indicated that response thresholds of IL-6-treated rats were significantly (*p *< 0.0001) less than those of SIF-treated rats. (**B**) Rats received dural administration of SIF (white bar, n = 17), 0.1 ng IL-6 (gray bar, n = 22) or 1 ng IL-6 (black bar, n = 16). Withdrawal thresholds to tactile stimuli were measured for 5 h and data were converted to area over the time-effect curve. IL-6 dose-dependently decreased the withdrawal threshold both in the face and in the hind-paw. Significant (*p < 0.05, **p < 0.01) differences among means for each group were determined by analysis of variance followed by Newman-Keuls post hoc test. (**C**) Application of 1 ng IL-6 was given with vehicle (white bars, 1% DMSO, n = 12) or with U0126 (black bars, 1 nmol, n = 12). Withdrawal thresholds to tactile stimuli were measured for 5 h and data were converted to area over the time-effect curve and normalized as a percentage of the IL-6-treated group. Coapplication of U0126 significantly abolished behavioral signs of tactile allodynia of the face and hind-paw (*p < 0.05, **p < 0.01).

A preclinical *in vivo *migraine model was used to evaluate the effect of meningeal IL-6 application on mechanical withdrawal thresholds both to the face and hindpaws [23]. Mechanical allodynia following dural stimulation is used as a surrogate for migraine as the majority of migraine patients experience cutaneous allodynia during the headache phase and allodynia is more common in migraine compared to other types of headaches [24,25]. Application of 1 ng IL-6 in SIF solution to the dura produced significant (*p *< 0.0001) time-dependent reductions in withdrawal thresholds to tactile stimuli applied to the face or the hind-paws compared with SIF application alone (Figure 1A). Maximal effects occurred 2 h after IL-6 application, and facial and hind-paw allodynia was present for at least 24 h (Figure 1A). IL-6 dose-dependently reduced the withdrawal thresholds compared with vehicle control as 1 ng IL-6 produced greater allodynia than that observed at 0.1 ng (Figure 1B). Co-application of the MEK inhibitor, U0126 (10 nmol) with 1 ng IL-6 prevented facial and hind-paw cutaneous allodynia (Figure 1C), indicating that IL-6 produces allodynia following dural application via activation of the MAP kinase (ERK) signaling pathway.

### Activation of the ERK pathway mediates IL-6-induced hyperexcitability of dural afferents

Nav1.7 is known to generate currents in response to slow ramp depolarization due to its slow inactivation kinetics, hence a ramp stimulus protocol was used to preferentially elicit activity of Nav1.7 [26]. Although this protocol elicits activation of Nav1.7 it should be noted that other sodium channels such as Nav1.8 may also be recruited as Nav1.7 and Nav1.8 are thought to work together in generating repetitive firing in sensory neurons [27,28]. Thus, this protocol likely produces firing via activation of multiple sodium channels but an increase in firing is nonetheless indicative of Nav1.7 sensitization.

**Figure 2:**
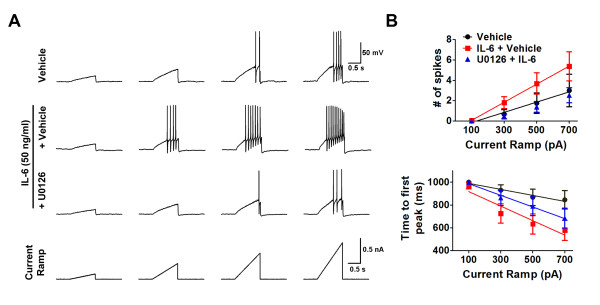
**IL-6 promoted ERK-dependent hyperexcitability of dural afferents in response to ramp current stimuli**. (**A**) Action potentials were elicited by 1 s ramp current injection ranging from 0.1 to 0.7 nA in 0.2 nA increments from resting membrane potential. Dural afferents treated with IL-6 showed increased numbers of action potentials and shorter time-to-first action potential (AP) peak compared with vehicle-treated dural afferents. IL-6-induced hyperexcitability was blocked by pretreatment with 10 μM U0126. (**B**) Difference in the mean numbers of action potentials among groups was analyzed by comparing the slopes and intercepts generated from linear regression. Comparison among several groups for time-to-first spike was performed by two-factor analysis of variance. Dural afferents treated with 50 ng/ml IL-6 (red square, n = 16) showed a significant (*p *< 0.05) increase in number of action potentials and a significant decrease in time-to-first peak compared with vehicle-treated dural afferents (black circle, n = 12). Pretreatment with 10 μM U0126 (blue triangle, n = 13) for 10 mins significantly reversed IL-6-induced hyperexcitability.

Retrogradely-labeled cells *in vitro *were selected for patch clamp experiments. Slow ramp currents from 0.1 to 0.7 nA with Δ = 0.2 nA were injected over 1 s (Figure 2A) to mimic slow depolarization. If cells fired in response to this protocol no further testing is done. If they did not fire with this protocol, a second protocol was run where the final ramp amplitude is 2 nA in 1 s. If cells fire in response to this protocol they were included in the data analysis as they technically responded to a ramp current injection but they are given 0 spikes for 0.1, 0.3, 0.5, and 0.7 nA since they did not fire in response to any of the slower ramps. If they did not fire in response to the 2 nA ramp they were excluded from analysis as they were determined to be cells that likely would not fire in response to a ramp. Dural afferents acutely treated with 50 ng/ml IL-6 for 15 min showed a significant increase in the number of spikes and a decrease in the latency to the first AP spike (Figure 2A and 2B), consistent with increased Nav1.7 activity. Pretreatment with 10 μM U0126 for 10 min significantly reversed the IL-6-induced increase in excitability (Figure 2A and 2B) indicating that, similar to IL-6 induced allodynia, these changes are due to activation of ERK signaling.

**Figure 3:**
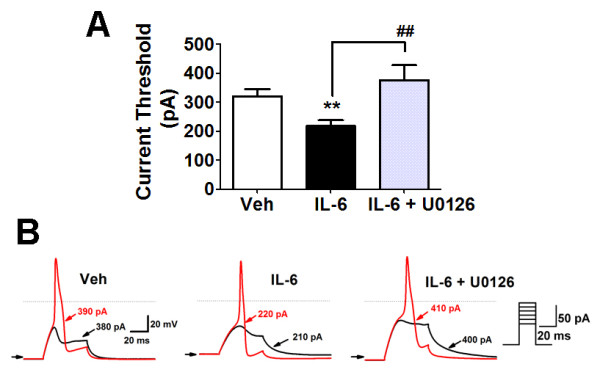
**IL-6 treatment significantly increased the dural afferent excitability, which was blocked by pretreatment with U0216**. (**A**) Current threshold was measured in dural afferents treated with vehicle (white bar, n = 30), 50 ng/ml IL-6 (black bar, n = 32) or 50 ng/ml IL-6 + 10 μM U0126 (gray bar, n = 13). Significant (**p < 0.01) differences among means for each group were determined by analysis of variance followed by Newman-Keuls post hoc test. Current threshold was significantly lowered after IL-6 treatment (**p < 0.01). Pretreatment with U0126 for 10 mins significantly reversed the hyperexcitability induced by IL-6 (^##^*p *< 0.01). (**B**) Action potentials were elicited by 25 ms step current injection from resting membrane potential. Horizontal line and arrow indicate 0 and -70 mV membrane potential, respectively. Current threshold for action potentials in the representative dural afferents treated with vehicle, 50 ng/ml IL-6 or 50 ng/ml IL-6 + 10 μM U0126.

Current clamp configuration was used to determine the current threshold, i.e. the minimum current required to initiate an action potential in the same group of dural afferents. Action potentials were elicited by injecting rectangular current steps (25 ms, Δ = 10 pA). The current threshold was significantly decreased for dural afferents acutely pretreated with IL-6 for 15 mins (217.4 ± 17.98 nA, n = 31) compared with dural afferents treated with vehicle (319.3 ± 25.14 nA, n = 30, **p < 0.01 vs vehicle) (Figure 3A and 3B). Although there was no depolarization of the resting membrane potential following IL-6 treatment, pretreatment with the MEK inhibitor U0126 significantly hyperpolarized resting membrane potentials (-67.24 ± 1.474 mV) compared with vehicle (-63.45 ± 0.7208, *p *< 0.05) or IL-6 treated neurons (-62.40 ± 0.7737 mV, *p *< 0.05). This finding is consistent with previous studies of Nav1.7 where U0126 treatment hyperpolarized resting membrane potentials [19]. Pretreatment with the MEK inhibitor U0126 for 10 mins reversed the IL-6-induced changes in current threshold (374.6 ± 52.45 nA, n = 13, ^##^*p *< 0.01 vs IL-6 alone), again indicating that IL-6 acts through the MAP kinase pathway.

### IL-6 treatment promotes direct association between ERK and Nav1.7

**Figure 4:**
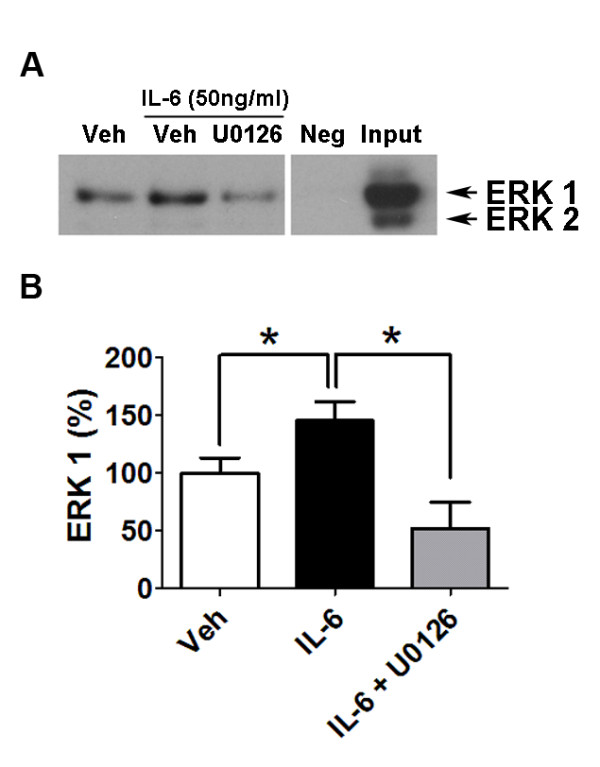
**IL-6 enhanced direct association between ERK1 and Nav1.7**. Western blot and quantification for tERK following 15 mins treatment of TG cultures with vehicle, IL-6 (50 ng/ml) or IL-6 (50 ng/ml) + U0126 (10 μM) (**A**) Interaction between ERK and Nav1.7 was examined by immunoprecipitation (IP) with antibody against Nav1.7 followed by immunoblotting (IB) for tERK. Omission of primary antibody was used as negative control and whole cell lysates were loaded as positive control. (**B**) ERK1 was quantified and values were expressed as percentage of vehicle. Significant (*p < 0.05) differences among means for each group were determined by analysis of variance followed by Newman-Keuls post hoc test.

To further explore whether IL-6-induced hyperexcitability of dural afferents was mediated through modulation of Nav1.7, we used a co-immunoprecipitation assay to determine direct associations between ERK and Nav1.7. In cells treated with IL-6 for 15 min, a significantly increased (*p < 0.05) amount of tERK was co-immunoprecipited with Nav1.7 compared to vehicle treatment (Figure 4A and 4B) although there was no change in the total level of Nav1.7 (data not shown). Pretreatment with the MEK inhibitor U0126 for 10 mins significantly reversed (*p < 0.05) the IL-6-induced increase in association between Nav1.7 and ERK. No signal was seen with cell lysates without primary antibody incubation (Figure 4A, Neg). In contrast to the observation that both tERK1 and tERK2 were detected in whole cell lysates (Figure 4A, Input), only tERK1 was detected in co-IP analysis (Figure 4A), consistent with a previous study showing that ERK1, but not ERK2, phosphorylated the L1 loop of Nav1.7 [19]. These data provide further evidence that IL-6-activated signaling pathways can regulate neuronal excitability through direct modulation of Nav1.7.

## Discussion

Understanding the endogenous processes that promote the activation and sensitization of meningeal nociceptors is important in explaining the mechanisms underlying migraine headache. The present findings provide direct evidence that IL-6 is important for sensitization of dural afferents by increasing neural excitability through modulation of Nav1.7. We also demonstrate that meningeal IL-6 application can produce migraine-like behavior through activation of the ERK pathway, supporting a role for IL-6 in migraine pathophysiology.

These studies demonstrate that direct meningeal application of exogenous IL-6 caused migraine-like behaviors in rats. However, the source of endogenous IL-6 during a migraine attack is not clear. Several lines of evidence have indicated that neurogenic inflammation underlies migraine headache pathogenesis with the involvement of at least 2 types of immune cells, dural mast cells and meningeal macrophages [29,30]. The meninges are densely populated with mast cells, which reside in close proximity to afferent endings mostly within the dura compared to other meningeal layers [31-33]. A variety of well-known migraine precipitants, including stress (via the release of corticotrophin releasing hormone CRH or factor CRF) and CGRP trigger mast cell degranulation and the subsequent release of their inflammatory mediators [29]. Relevant to the studies described here are reports that human mast cells can release IL-6 following stimulation [34,35]. In addition to mast cells, IL-6 released from dural macrophages may also contribute to stress-induced neurogenic inflammation [30]. Regardless of the source, IL-6 has the ability to sensitize nociceptors through actions on TRPV1 and ERK-mediated signaling to translation machinery [15,36]. The experiments described here demonstrated that IL-6 application was able to sensitize identified dural afferents, and suggested additional mechanisms of IL-6 induced sensitization through phosphorylation of sodium channels.

Human genetic studies have demonstrated an important role for the sodium channel Nav1.7 in pain [37]. Gain-of-function mutations of Nav1.7 are directly linked with several extreme pain conditions in humans such as erythromelalgia and paroxysmal extreme pain disorder, whereas loss-of-function mutation of Nav1.7 is associated with congenital insensitivity to pain [37]. Although the gain-of-function mutations do not lead to headache and the location specific nature of the spontaneous pain in these disorders is poorly understood, these conditions highlight the importance of this channel in nociceptive signaling and suggest that sensitization of Nav1.7 may contribute to enhanced pain signaling from many areas including the head. Due to its distinctive slow development of closed-state inactivation, Nav1.7 is able to generate current in response to sub-threshold depolarization, thus playing an important role in amplifying excitatory inputs and modulating neuronal excitability [26]. Additionally, inhibition of Nav1.7 is known to decrease neuronal excitability [38,39]. Preclinical work has also indicated an important role for Nav1.7 in mediating inflammatory pain as supported by the evidence that formalin-induced mechanical allodynia and thermal hyperalgesia are abrogated in Nav1.7 knockout mice [40]. Moreover, mRNA and protein levels of Nav1.7 increase following carrageenan injection, which parallel the increase in TTX-S currents [41]. Hence, preclinical and clinical studies have created a compelling rationale for targeting Nav1.7 in inflammatory pain. The present work indicates that IL-6 application increases the number of spikes and decreases the latency to the first AP in response to ramp stimuli protocols, which are consistent with hyperexcitability induced by Nav1.7 phosphorylation [19]. This IL-6-induced hyperexcitability is mediated through ERK signaling, which is similar to prior work showing that inhibition of ERK1/2 decreases excitability in DRG neurons [19]. Additionally, and consistent with the previous study showing that pERK1, but not pERK2 phosphorylated the L1 loop of Nav1.7 [19], increased association between ERK1 and Nav1.7 was detected following IL-6 treatment, indicating that IL-6-activated signaling pathways are capable of modulating Nav1.7 directly. While we cannot rule out the possibility that modulation of other channels contributes to electrophysiological effects following IL-6 treatment, the findings reported here support the hypothesis that IL-6 produces sodium channel-dependent hyperexcitability and migraine-related behavior through activation of the ERK pathway.

## Conclusions

This study provides direct evidence that IL-6 can sensitize dural afferents in a manner consistent with sodium channel phosphorylation and that it produces prolonged migraine-related pain behavior through activation of the ERK pathway. Although there is currently no direct link between Nav1.7 and migraine, these findings suggest that an IL-6/Nav1.7 signaling axis can be an important mediator of headache pain and that drugs targeting IL-6 signaling may have efficacy in the treatment of migraine headache.

## Competing interests

The authors declare that they have no competing interests.

## Authors' contributions

JY, OKM, TJP and GD conceived of the study and designed experiments, JY and OKM performed experiments. JY and OKM analyzed data. JY and GD wrote the manuscript. All authors read and approved the final manuscript.
